# The Physiological Response Mechanism of Peanut Leaves under Al Stress

**DOI:** 10.3390/plants13121606

**Published:** 2024-06-10

**Authors:** Jianning Shi, Jianyu Li, Yuhu Pan, Min Zhao, Rui Zhang, Yingbin Xue, Ying Liu

**Affiliations:** 1 Department of Biotechnology, College of Coastal Agricultural Sciences, Guangdong Ocean University, Zhanjiang 524088, China; 2 Department of Agronomy, College of Coastal Agricultural Sciences, Guangdong Ocean University, Zhanjiang 524088, China

**Keywords:** peanut, Al toxicity, leaf growth, ionomics, hormonomics

## Abstract

Aluminum (Al) toxicity in acidic soils can significantly reduce peanut yield. The physiological response of peanut leaves to Al poisoning stress still has not been fully explored. This research examined the influences of Al toxicity on peanut leaves by observing the leaf phenotype, scanning the leaf area and perimeter, and by measuring photosynthetic pigment content, physiological response indices, leaf hormone levels, and mineral element accumulation. Fluorescence quantitative RT–PCR (qPCR) was utilized to determine the relative transcript level of specific genes. The results indicated that Al toxicity hindered peanut leaf development, reducing their biomass, surface area, and perimeter, although the decrease in photosynthetic pigment content was minimal. Al toxicity notably affected the activity of antioxidative enzymes, proline content, and MDA (malondialdehyde) levels in the leaves. Additionally, Al poisoning resulted in the increased accumulation of iron (Fe), potassium (K), and Al in peanut leaves but reduced the levels of calcium (Ca), manganese (Mn), copper (Cu), zinc (Zn), and magnesium (Mg). There were significant changes in the content of hormones and the expression level of genes connected with hormones in peanut leaves. High Al concentrations may activate cellular defense mechanisms, enhancing antioxidative activity to mitigate excess reactive oxygen species (ROS) and affecting hormone-related gene expression, which may impede leaf biomass and development. This research aimed to elucidate the physiological response mechanisms of peanut leaves to Al poisoning stress, providing insights for breeding new varieties resistant to Al poisoning.

## 1. Introduction

The ongoing expansion of human production activities has led to a growing environmental impact from acid deposition [1]. Intensive agricultural practices and acidic precipitation contribute to the accumulation of nitrogen and sulfur deposits in soil, resulting in a decrease in the soil pH [2]. The presence of acidic soils is detrimental to crop production [3]. Global data indicate that approximately 30% of the world’s land is categorized as acidic soil [4]. In China, acidic soil currently comprises approximately 22.7% of the country’s cultivated land [5]. Moreover, soil acidity in China is experiencing a rapid increase [6]. As soil acidification becomes more pronounced, the dissolution of aluminum (Al) in soil intensifies, thereby becoming a prominent factor that limits plant growth in acidic soil conditions [7]. Al is the most abundant metal on Earth and can exist in various forms in acidic soils, including Al^3+^, Al(OH)^2+^, Al(OH)_2_^+^, and Al(OH)_4_^−^ [7,8,9]. Superabundant Al^3+^ has the most severe negative effect on plants [7,8,9]. After being subjected to Al poisoning, the content of Al^3+^ in the cytoderm and the cytosol increases, resulting in the inhibition of cell elongation and division [10]. Rice (*Oryza sativa* L.) cells exposed to Al toxicity display all kinds of chromosome aberrations and cell fission irregularities [10]. An increase in reactive oxygen species (ROS) may be induced by the excessive accumulation of Al in plants, resulting in lipid peroxidatic reactions and the dysfunction of plasma membrane organelles [11]. To mitigate oxidative damage, plants promote the enzyme activities of APX (ascorbate peroxidase), CAT (catalase), SOD (superoxide dismutase), and POD (peroxidase) to eliminate excess ROS [12]. Furthermore, soluble sugars, soluble proteins, and proline assist in osmotic regulation to maintain normal cell growth [12]. Maize (*Zea mays* L.) leaves exhibit increased SOD and CAT activities after exposure to Al toxicity stress, indicating oxidative damage [13]. Similarly, in buckwheat (*Fagopyrum esculentum* Moench) roots subjected to Al toxicity stress, there is a noticeable increase in APX, POD, CAT, SOD, soluble sugars, soluble proteins, proline, and malondialdehyde (MDA) content [14].

The roots of plants are the first to be affected by Al toxicity stress [15]. Consequently, there have been more studies on the response of plant roots to Al toxicity stress than on the impact on plant leaves [15]. The adverse influences of Al poisoning stress on plant leaves primarily involve a reduction in chlorophyll content, photosynthetic capacity, and nutrient content [15]. Furthermore, Al poisoning stress disrupts the photosystem and diminishes pigment content, bringing about decreasing leaf photosynthetic efficiency [16]. For instance, one Al-sensitive rye (*Secale cereale* L.) variety displays chloroplast enlargement and increased Al accumulation under Al poisoning stress, which causes a reduction in the content of chlorophyll and impairs photosynthesis [16]. In maize, sorghum (*Sorghum bicolor* (L.) Moench), and most Al-sensitive plants, even low concentrations of Al inhibit leaf elongation and impair the plant’s photosynthetic ability [17,18]. Numerous genes associated with Al tolerance have been identified in plant leaves [19,20]. In tea (*Camellia sinensis* (L.) O. Kuntze), genes involved in cell wall function, reactive oxygen species, cell transport, and signal transduction have a role in the response to excessive Al [21]. Additionally, under Al poisoning stress, the brassinolide content in maize leaves is influenced by tricarboxylic acid cycle (TCA) circulating enzymes [22].

Peanut (*Arachhis hypogaea* L.) is a crucial source of fat and protein [23]. It has an important part in ensuring the supply of oils and proteins and promoting the stable development of agriculture [23]. Currently, China’s peanut production area is nearly 5 million hectares, with an output of about 20 million tons, which significantly contributes to the efficient supply of plant oils [24]. Results have demonstrated that Al poisoning stress significantly inhibits the growth and development of peanuts. After 14 days of 5 mM Al treatment, the peanut plant height was lower by 40% as compared with the control [25]. Nonetheless, the physiological response mechanism of peanut leaves under Al poisoning stress remains unclear.

Plant hormone regulation is a vital mechanism for plants to regulate growth and to adapt to external stress [26]. Exogenous regulatory factors (EARFs), such as nutrients and plant hormones, can help alleviate the symptoms of high Al stress in plants [27]. Phytohormones, including cytokinin, JA (jasmonic acid), ABA (abscisic acid), GA (gibberellic acid), and SA (salicylic acid), are responsible for the elongation, differentiation, and proliferation of plant cells [28]. Cytokinin increases the leaf wrinkling of lettuce (*Lactuca sativa* L.), whereas gibberellin flattens lettuce leaves [29]. The hormone-regulating gene *LsKN1* influences the waviness of lettuce leaves, with upregulated *LsKN1* causing an increase in cytokinin biosynthesis and a decrease in gibberellin biosynthesis [29]. In lemon (*Citrus*×*Limonia osbeck*), Al toxicity stress directly induces ABA biosynthesis in leaves, affecting leaf hydration [30]. In soybeans, *GmNIMIN1* and other genes are crucial for the response to salicylic acid synthesis, whereas *GmBPI1* and other genes assist in the response to jasmonic acid synthesis, helping soybeans to adapt to abiotic stress [31]. In addition, indole-3-acetic acid (IAA) is the major growth hormone in plants, regulating plant growth, development, and response to adversity [32]. In white clover (*Trifolium repens* L.), TrIAA18 has been shown to be involved in the positive regulation of Al, drought, and salt stress, as well as in the interaction with TrARF5, indicating that TrIAA18 may regulate abiotic stress responses by affecting TrARF5 [32]. Although phytohormones have a pivotal role in peanut growth, the regulation mechanism of phytohormones’ effects on the growth of peanut leaves suffering from Al poisoning stress has not been thoroughly studied.

The gene family involved in metal ion transport plays a critical role in the assimilation of nutrient substances in plants [33]. For instance, the natural-resistance-associated macrophage protein (NRAMP) family participates in metal absorption and transportation processes in plants [33]. The expression levels of *GmNRAMP1a* and *GmNRAMP5a* in soybeans (*Glycine max* L.) are upregulated under excessive Cu^2+^ treatment [34]. Metal tolerance proteins (MTPs) are involved in the transportation of metal ions in plants [35]. When exposed to Cd^2+^, Co^2+^, Fe^2+^, Mn^2+^, and Zn^2+^ treatments, the transcriptional level of *MTP1.1* in leaves of soybean significantly increases [36]. Current research has paid close attention to the functions of the MTP and NRAMP families in inducing divalent metal ions such as Fe^2+^ and Cd^2+^ [33,35].

Since roots are the first part of plants affected by aluminum toxicity stress, most current studies focus on the mechanism of aluminum resistance in plant roots [15]. However, there are few studies on the effects of aluminum toxicity stress on plant leaves [15]. The leaf of the peanut plant is the primary site for synthesizing organic matter through photosynthesis. However, the physiological response mechanism of peanut leaves to Al poisoning stress remains unclear, and the physiological regulatory pathway of peanut leaves in response to Al poisoning stress needs to be investigated. To study the role of peanut leaves in the resistance of peanut plants to Al poisoning, the physiological and molecular bases of peanut leaves’ response to Al toxicity stress were explored. The objective of this research was to explore the physiological response mechanism of peanut leaves to Al poisoning stress and to lay a basic foundation for cultivating new peanut varieties resistant to Al poisoning.

## 2. Results

### 2.1. Influences of Al Toxicity Stress on Peanut Leaf Growth

**Figure 1:**
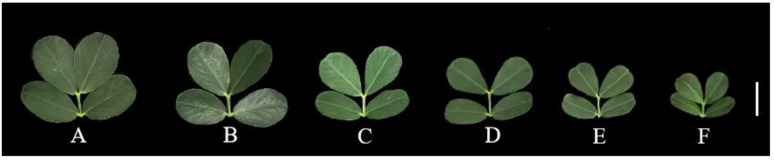
Peanut leaf phenotype under different Al concentrations. Peanuts were treated with different concentrations of Al for 20 days. (**A**) 0, (**B**) 0.5, (**C**) 1, (**D**) 2, (**E**) 4, and (**F**) 8 mM Al (bar = 2 cm).

**Figure 2:**
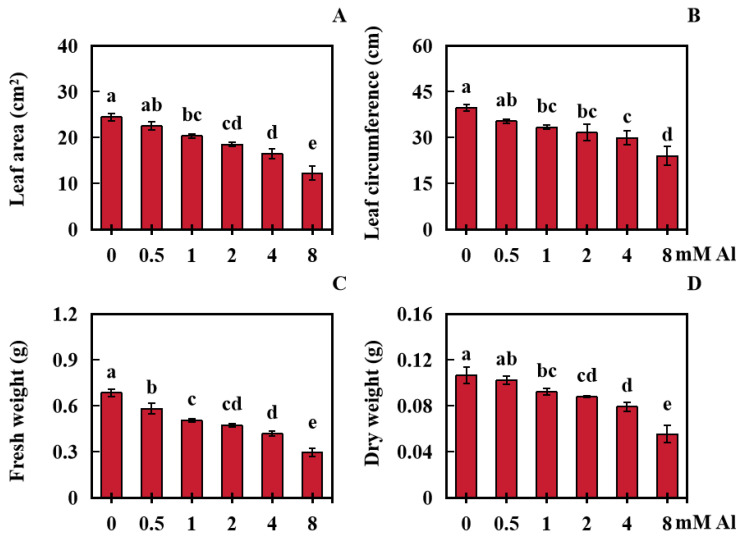
Dry and fresh weights of peanut leaves and leaf sizes treated with different Al concentrations. (**A**) Leaf area; (**B**) Blade circumference; (**C**) Leaf fresh weight; (**D**) Leaf dry weight. The results were displayed as the average value (±) SD (standard deviation) of three repeated experiments. The Waller–Duncan multiple comparison test was adopted for comparing the differences in Al toxicity among all the groups. Different lowercase letters marked on the column diagrams indicate where there were significant differences between the data (*p* < 0.05).

The effects of Al poisoning stress on peanut leaf biomass were investigated under different Al treatment concentrations. The results indicated that Al poisoning stress had a negative effect on peanut leaf biomass (Figure 1A–F and Figure 2A–D). The leaf area and girth decreased with increasing Al concentration, and the dry and fresh weights of the peanut leaves also decreased with increasing Al concentration (Figure 1A–F and Figure 2A–D).

Compared with the control (0 mM Al), the peanut leaf area in the 0.5 mM Al treatment group was not markedly reduced. However, the peanut leaf area of in the other treatment groups (1, 2, 4, and 8 mM Al) was significantly reduced by 16.94%, 24.45%, 32.69%, and 49.99%, respectively (Figure 2A). In terms of leaf circumference, that of peanut leaves in the 0.5 mM Al treatment group did not markedly decrease, whereas those in the other treatment groups significantly decreased by 15.99%, 20.41%, 24.64%, and 39.38%, respectively (Figure 2B). Compared with those in the control, the fresh weights of leaves in the treatment groups (0.5, 1, 2, 4, and 8 mM Al) were significantly lower by 14.96%, 26.18%, 30.81%, 39.02%, and 56.80%, respectively (Figure 2C). Similarly, compared with those in the control, the dry weight of the peanut leaves in the 0.5 mM Al treatment group did not significantly decrease, whereas those in the other treatment groups (1, 2, 4, and 8 mM Al) decreased significantly by 13.67%, 17.63%, 25.77%, and 48.11%, respectively (Figure 2D).

### 2.2. Influences of Al Poisoning Stress on Chlorophyll and Total Carotenoid Contents in Peanut Leaves

**Figure 3:**
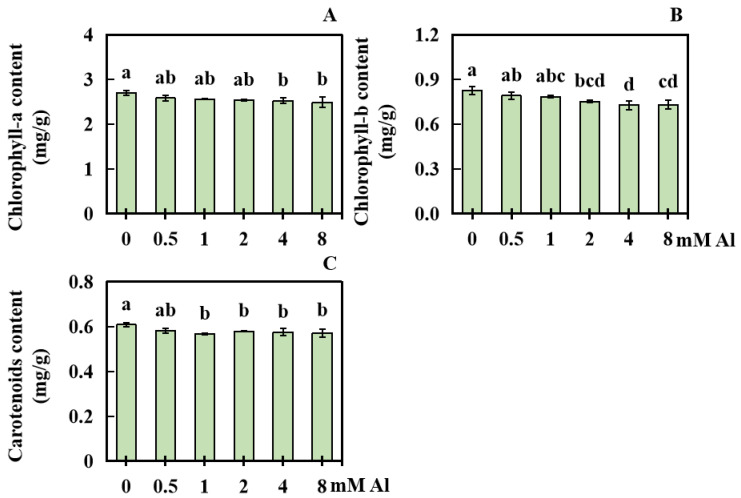
Changes in the photosynthetic pigment content in peanut leaves under different Al concentrations. The content of (**A**) chlorophyll-a; (**B**) chlorophyll-b; and (**C**) carotenoids in peanut leaves was determined. The results were displayed as the average value (±) SD (standard deviation) of three parallel experiments. The data were based on the fresh mass. The Waller–Duncan multiple comparison test was adopted to compare the differences in Al toxicity among all the groups. Different lowercase letters marked on the column diagrams indicate where there were significant differences between the data (*p* < 0.05).

After treatment with Al, the photosynthetic pigment content in peanut leaves experienced some degree of impact. Compared with the control (0 mM Al), the chlorophyll-a content in peanuts subjected to low-concentration Al treatment did not significantly decrease. However, the chlorophyll-a content in peanuts subjected to 4 or 8 mM Al treatment significantly decreased by 6.33% and 7.75%, respectively (Figure 3A). Similarly, the content of chlorophyll-b in peanuts treated with lower Al concentrations did not significantly decrease, whereas the content of chlorophyll-b in peanuts treated with 2, 4, and 8 mM Al significantly decreased by 8.80%, 11.84%, and 11.34%, respectively (Figure 3B). Compared with the control, the carotenoid content in peanut leaves subjected to 1, 2, 4, and 8 mM Al treatment decreased by 6.82%, 4.73%, 5.38%, and 6.06%, respectively (Figure 3C).

### 2.3. Effects of Al Poisoning Stress on Various Physiological Parameters of Peanut Leaves

**Figure 4:**
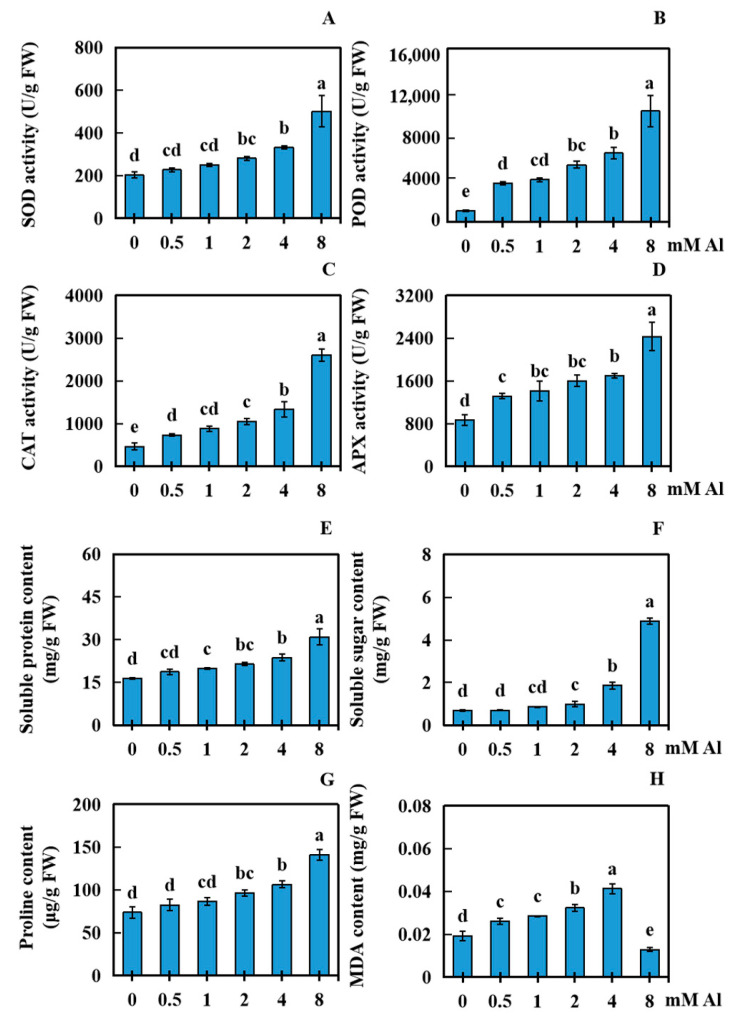
Effect on various physiological indices in peanut leaves subjected to Al poisoning stress. Peanut leaves were treated with exogenous Al for 20 days at concentrations of 0, 0.5, 1, 2, 4, and 8 mM. Eight physiological indices were measured: the activities of (**A**) SOD, (**B**) POD, (**C**) CAT, and (**D**) APX and the content of (**E**) soluble proteins, (**F**) soluble sugars, (**G**) proline, and (**H**) MDA. The results were displayed as the average value (±) SD (standard deviation) of three parallel experiments. The Waller–Duncan multiple comparison test was adopted to compare the differences in Al toxicity among all the groups. Different lowercase letters marked on the column diagrams indicate where there were significant differences between the data (*p* < 0.05).

When peanuts were suffering from Al toxicity, the physiological response indices of peanut leaves were significantly affected. Using varying concentrations of Al (0.5, 1, 2, 4, and 8 mM) for 20 days showed that the increase in Al concentration had a significant impact on the physiological response of peanut leaves. Compared with the control (0 mM), the activity of SOD in the 0.5 and 1 mM treatment groups did not markedly differ, whereas in the 2, 4, and 8 mM treatment groups, the SOD activity increased by 38.73%, 63.66%, and 147.80%, respectively (Figure 4A). The activity of POD in all the treatment groups (0.5, 1, 2, 4, and 8 mM Al) increased significantly, by 244.11%, 276.12%, 408.16%, 512.20%, and 884.31%, respectively (Figure 4B). CAT activity in all the treatment groups (0.5, 1, 2, 4, and 8 mM Al) increased significantly, by 57.14%, 89.29%, 125.00%, 185.71%, and 457.14%, respectively (Figure 4C). The activity of APX in all the treatment groups significantly increased by 51.92%, 63.46%, 84.62%, 96.15%, and 180.77%, respectively (Figure 4D). Compared with the control, the content of soluble protein in the 0.5 mM treatment group did not significantly vary, whereas the content of soluble protein in the other treatment groups (1, 2, 4, and 8 mM Al) increased significantly, by 21.34%, 30.29%, 44.83%, and 88.52%, respectively (Figure 4E). The content of soluble sugars in the 0.5 and 1 mM treatment groups did not significantly vary, whereas in the 2.0, 4.0, and 8.0 mM treatment groups, the soluble sugar content significantly increased by 44.49%, 171.89%, and 609.61%, respectively (Figure 4F). The proline content did not significantly change in the 0.5 and 1 mM treatment groups, whereas it significantly increased by 30.98%, 44.58%, and 91.76% in the 2.0, 4.0, and 8.0 mM treatment groups, respectively (Figure 4G). The content of MDA in the treatment groups (0.5, 1, 2, and mM Al) significantly increased by 35.51%, 48.44%, 68.20%, and 115.04%, respectively; however, the MDA level in the 8.0 mM treatment group significantly decreased by 32.88% (Figure 4H).

### 2.4. Effects of Al Toxicity Stress on the Absorption of Ten Elements in Peanut Leaves

**Figure 5:**
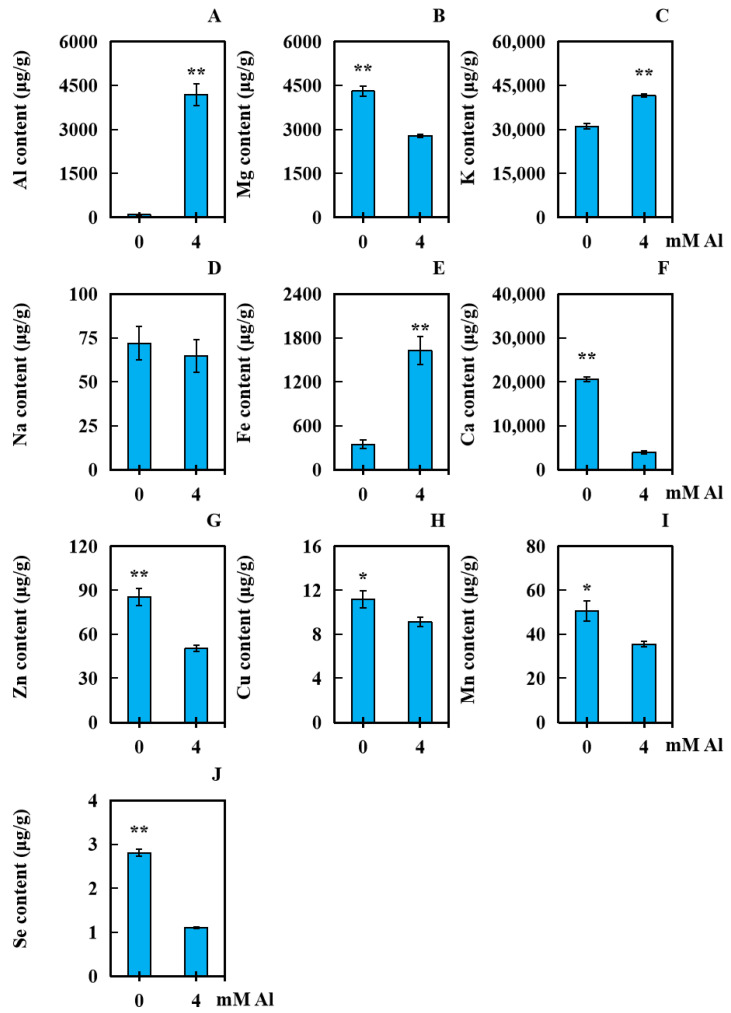
The contents of ten kinds of elements in peanut leaves subjected to Al poisoning stress. The contents of 10 elements ((**A**): Al; (**B**): Mg; (**C**): K; (**D**): Na; (**E**): Fe; (**F**): Ca; (**G**): Zn; (**H**): Cu; (**I**): Mn; and (**J**): Se) were determined. The results were displayed as the average value (±) SD (standard deviation) of three parallel experiments. Independent sample *t* tests were adopted to compare the differences between the control and the Al toxicity groups. Different asterisks (*) marked on the column diagrams indicate where there were significant differences between the data (* *p* < 0.05; ** *p* < 0.01).

With 0 mM Al as the control, samples were obtained after 20 days of applying 4 mM exogenous Al (Al toxicity group). The contents of Fe, K, Zn, Se, Na, Cu, Mg, Ca, Mn, and Al were analyzed to investigate the effect of Al poisoning stress on the accumulation of these 10 elements in leaves of the peanut plant The accumulation of Al, K, and Fe significantly increased by 9141.23%, 33.63%, and 367.06%, respectively (Figure 5A,C,E). No significant change in the accumulation of Na in the peanut leaves was observed compared to that in the control (Figure 5D). The contents of Cu and Mn decreased significantly, by 18.39% and 29.85%, respectively (Figure 5H,I). Additionally, the accumulations of Mg, Ca, Zn, and Se reduced markedly, by 35.20%, 80.70%, 41.07%, and 60.55%, respectively (Figure 5B,F,G,J).

### 2.5. Influences of Al Poisoning Stress on Six Kinds of Hormone Levels in Peanut Leaves 

**Figure 6:**
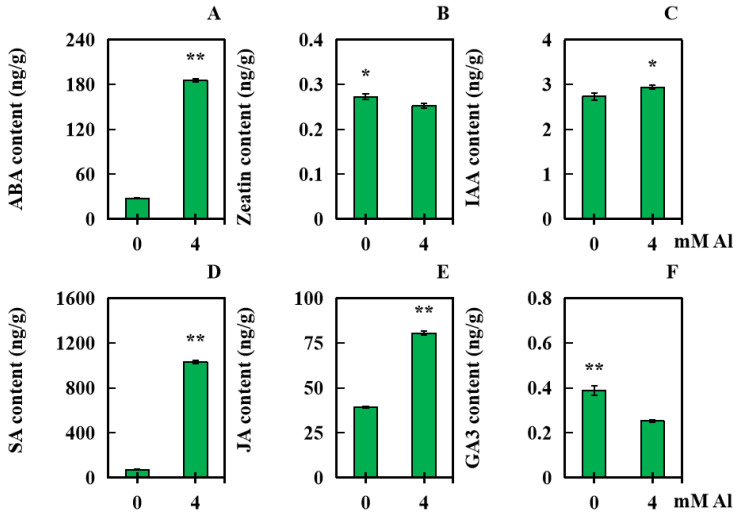
Six kinds of hormone levels in peanut leaves under Al toxicity stress. The contents of six kinds of hormones ((**A**): ABA; (**B**): Zeatin; (**C**): IAA; (**D**): SA; (**E**): JA; and (**F**): GA_3_) in peanut leaves were determined after being subjected to Al poisoning stress. The results were displayed as the average value (±) SD (standard deviation) of three parallel experiments. Independent sample *t* tests were adopted to compare the differences between the control and Al toxicity groups. Different asterisks (*) marked on the column diagrams indicate where there were significant differences between the data (* *p* < 0.05; ** *p* < 0.01).

With 0 mM Al as the control, leaf samples were obtained from the peanut plant after applying 4 mM exogenous Al for 20 days. The levels of six kinds of hormone (Zeatin, JA, SA, IAA, ABA, and GA_3_) were analyzed to explore the effect of Al poisoning stress on hormone accumulation in peanut leaves. Compared with those in the controls, the indoleacetic acid (IAA) levels in the treated plants increased significantly by 7.52% (Figure 6C). The levels of ABA, SA, and JA increased significantly—in order, by 569.15%, 1320.90%, and 104.83% (Figure 6A,D,E). However, the level of Zeatin decreased significantly, by 7.32% (Figure 6B). The content of GA_3_ decreased significantly, by 34.82% (Figure 6F).

### 2.6. Analysis of Related Gene Expression Levels in Peanut Leaves Subjected to Al Poisoning Stress

**Figure 7:**
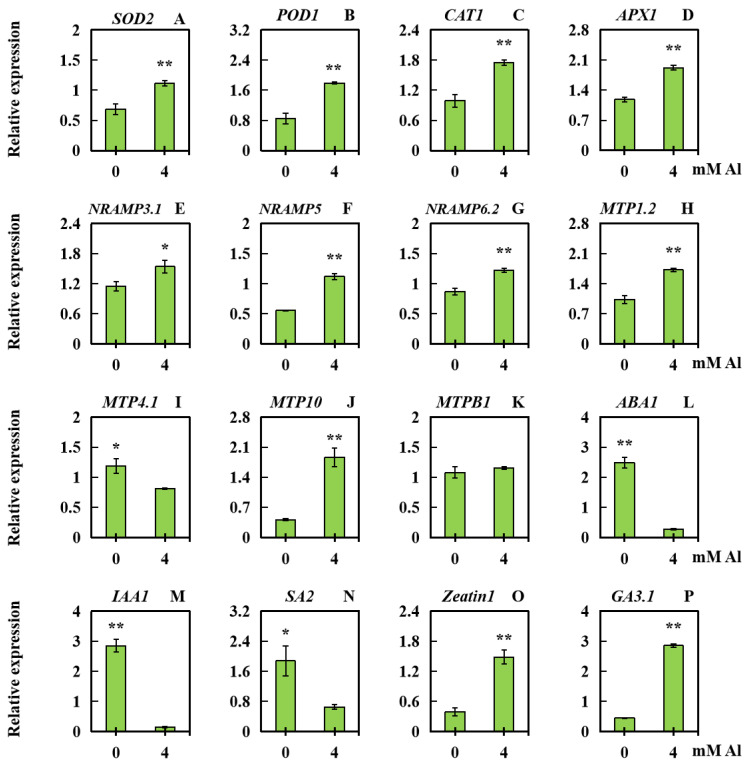
The expression levels of 16 related genes in peanut leaves subjected to Al poisoning stress. (**A**) SOD2 (superoxide dismutase 2); (**B**) POD1 (peroxidase 1); (**C**) CAT1 (catalase 1); (**D**) APX1 (ascorbate peroxidase 1); (**E**) Nramp3.1 (natural-resistance-associated macrophage protein 3.1); (**F**) Nramp5 (natural-resistance-associated macrophage protein 5); (**G**) Nramp6.2; (**H**) MTP1.2 (metal tolerance protein 1.2); (**I**) MTP4.1; (**J**) MTP10; (**K**) MTPB1; (**L**) ABA1 (abscisic acid 1); (**M**) IAA1 (indoleacetic acid 1); (**N**) SA2 (salicylic acid 2); (**O**) Zeatin1; (**P**) GA3.1 (Gibberellin 3.1). The results were displayed as the average value (±) SD (standard deviation) of three parallel experiments. Independent sample *t* tests were adopted to compare the differences between the control and Al toxicity groups. Different asterisks (*) marked on the column diagrams indicate where there were significant differences between the data (* *p* < 0.05; ** *p* < 0.01).

In order to examine the effect of Al poisoning stress on the expression levels of related genes in leaves, the relative expression levels of three categories of genes were quantified via qPCR (quantitative RT-PCR). These categories included genes associated with physiological responses (*SOD2*, *POD1*, *CAT1*, and *APX1*), ion transport (*Nramp3.1*, *Nramp5*, *Nramp6.2*, *MTP1.2*, *MTP4.1*, *MTP10*, and *MTPB1*), and genes related to hormones (*IAA1*, *ABA1*, *SA2*, *GA3.1*, and *Zeatin1*). *SOD2*, *POD1*, *CAT1*, *APX1*, *Nramp3.1*, *Nramp5*, *Nramp6.2*, *MTP1.2*, *MTP10*, *Zeatin1*, and *GA3.1* exhibited upregulated expression under Al poisoning stress (Figure 7A–H,J,O,P), whereas *MTP4.1*, *ABA1*, *IAA1*, and *SA2* showed downregulated expression (Figure 7I,L,M,N). There was no significant change in *MTPB1* expression (Figure 7K).

## 3. Discussion

Al^3+^ is recognized as the primary factor inhibiting plant growth in acidic soils [37]. In this study, peanut leaves treated with 0 mM Al were used as the control. Under severe Al toxicity stress, the fresh weight, dry weight, leaf area, and leaf circumference of peanut leaves significantly decreased, indicating substantial inhibition of peanut leaf growth.

Al has been shown to have various impacts on photosynthesis. Previous studies have revealed phenomena such as the inhibition of pigment biosynthesis, changes in enzymes related to the Calvin cycle, and alterations in chloroplast structure [16]. Al toxicity can lead to morphological changes in chloroplasts, including increased size, stroma compression, disorder in the lamellar system, and rupture of the thylakoid membrane [16]. Degradation of thylakoid membranes induced by Al is often associated with damage to photosystem II (PSII), a reduction in the PSII electron transport rate, and the shutdown of PSII reaction centers [16].

In duckweed (*Lemna minor* L.), the levels of chlorophyll a, chlorophyll b, and carotenoids were found to decrease by 27%, 20%, and 21%, respectively, compared with those in the control group when exposed to 0.3 mM Al [38]. Al stress significantly damages the electron transport chain from PSII to PSI, reducing the photochemical efficiency and increasing the production of ROS (reactive oxygen species) [38]. Similar impacts, such as a reduction in chlorophyll a and b content, have also been observed in Al-sensitive varieties of rye (*Secale cereale* L.) under Al toxicity stress [15]. Moreover, micromolar concentrations of Al present in the soil are enough to induce several irreversible toxicity symptoms such as the rapid and transient over-generation of reactive oxygen species (ROS) such as superoxide anion, hydrogen peroxide, and hydroxyl radical, resulting in oxidative bursts [39]. In addition, under heavy-metal stress, plants undergo changes in their molecules, physiology, and morphology [40,41]. Physiological responses such as those involving malondialdehyde (MDA), superoxide dismutase (SOD), peroxidase (POD), and proline (PRO) may alleviate the toxicity of heavy metals by regulating osmosis or by scavenging reactive oxygen species (ROS) [42]. After being subjected to 15 mg/L Cd stress for 5 days, SOD activity in *Pistia stratiotes* L. decreased significantly, while CAT activity increased significantly, indicating that the plants themselves reduce membrane lipid peroxidation and reactive oxygen accumulation mainly by adjusting the activity of antioxidative enzymes [43]. Similarly, excessive Al can also cause changes in the activity of antioxidative enzymes in plants [44]. For example, SOD, POD, and CAT activities of aluminum-resistant rice (CNA-1158) increase significantly after 9 days of 1 mM Al treatment, and ROS in cells are consumed directly or indirectly by antioxidative enzymes [44].

Furthermore, it has been suggested that one explanation for changes in the photosynthetic organ activity could be the interference of Al in the absorption of iron and manganese by plants [16,45,46]. Mn is essential for maintaining the oxygen-evolving complex in PSII, whereas Fe is a component of PSII, PSI, the cytochrome b6-f complex, and ferridoxin [45,46]. X-ray spectroscopy in rye has shown low levels of Mg, Fe, and Mn, particularly in the chloroplasts of sensitive genotypes [16]. In the present study, changes in the levels of Mn, Fe, and Mg caused by Al might also be factors influencing the photosynthetic capacity of peanuts.

However, it should be noted that in this study, although Al toxicity stress had a certain influence on the levels of chlorophyll b, chlorophyll a, and carotenoids in peanut leaves, the effect of even high concentrations of Al on photosynthetic pigments in peanut leaves was relatively limited. This suggests that the photosynthetic system of peanut leaves may have a high tolerance for Al toxicity stress.

After being subjected to aluminum toxicity stress, the equilibrium between the output and degradation of ROS and free radicals in plants is disrupted [47]. This disruption leads to enzyme deactivation, oxidative stress damage, and cell membrane peroxidization [47]. The osmotic adjustment system and antioxidative defense system play crucial parts in plant resistance to oxidative stress response [48]. Plants create antioxidants such as APX, CAT, SOD, and POD to mitigate oxidative damage [48]. Additionally, compounds such as proline, soluble proteins, and soluble sugars facilitate the regulation of the cell osmotic potential and reduce the water loss caused by external stress [48]. The concentration of MDA serves as an important reference index for measuring the extent of oxidative damage to plant membrane lipids [49]. Therefore, assessing the activities of antioxidants and the presence of osmotic adjustment substances can provide valuable insights into the cell’s metabolic activity and overall health status.

Plant SOD family genes are mainly classified into three categories on account of their metal accessory factors: FeSOD, Cu/ZnSOD, and MnSOD [50]. SOD has an essential role as the initial defense mechanism of the antioxidative system in plants [50]. Among the members of *Brassica napus* L., *BnFeSOD5*, a member of the SOD gene family, exhibits the highest expression level in the leaves [51]. Moreover, it is significantly upregulated under various abiotic stresses (salt, cold, waterlogging, and drought) as well as under ABA, GA, and IAA treatments [51]. Tomato (*Solanum lycopersicum* L.) also shows relatively high expression of *SlSOD1*, a member of the SOD gene family, in the leaves. The expression level of *SlSOD1* in leaves is notably upregulated when plants are exposed to salt stress [52]. In our study, the differential absorption of Cu, Zn, Mn, and Fe induced by Al^3+^ may lead to varying expression patterns of the peanut *SOD1* gene, subsequently affecting SOD activity in peanut leaves. The plant POD gene family, also known as the PRX family, is responsible for guiding the synthesis of specific REDOX enzymes known as class III peroxidases [53]. Notably, research has shown that the expression of *PRX38* in sugarcane (*Saccharum officinarum* L.) leaves significantly increases after Cd^2+^ treatment [54]. This suggests that Class III PRX (POD) genes enhance the resistance of sugarcane to Cd stress by activating the antioxidative system and removing reactive oxygen species [54]. In our study, Al-induced differential expression of the *POD* gene resulted in increased POD activity in peanut leaves, thereby affecting the antioxidative capacity of peanuts. Rice (*Oryza sativa* L.) has been found to possess three *CAT* family members, with *OsCAT3* exhibiting the highest expression in the stems and leaves of rice [55]. Knocking out *OsCAT3*_crispr_ using CRISPR technology in transgenic rice lines resulted in growth restriction, plant dwarfing, and leaf bleaching. Additionally, the activities of the CAT, SOD, and POD antioxidative enzymes significantly decreased [55]. In our study, high concentrations of Al caused differential expression of the *CAT* gene, subsequently affecting CAT activity. Overexpression and transformation of the *AtApx1* gene isolated from *Arabidopsis thaliana* (L.)Heynh. into brassica (*Brassica juncea* (L.) Czern.) led to increased APX activity and decreased H_2_O_2_ and MDA levels in the leaves of the overexpressing wild-type plants under salt stress [56]. This indicates that the *AtApx1*-overexpressing lines experience less oxidative damage and that AtApx1 plays a crucial role in maintaining ROS homeostasis and enhancing plant stress resistance under salt stress [56]. Our study suggested that the differential expression of APX-related genes mediated by Chalco might be responsible for the increased APX activity in peanut leaves. Osmoregulatory substances such as soluble sugars, soluble proteins, and proline in plant cells play a vital role in maintaining the cellular osmotic balance, alleviating the physiological and metabolic imbalances caused by stress and preserving cell structure stability [57]. Our study revealed that the levels of soluble proteins, soluble sugars, and proline in peanut leaves tended to increase, indicating that peanut leaves can resist Al stress by increasing the amount of osmotic regulatory substances. MDA is a coproduct of lipid peroxidation (LPO) resulting from oxidative injury, which mirrors the degree of membrane injury caused by peroxidation [58]. Previous studies have shown that Al-treated *Pinus massoniana* Lamb. exhibits an increase in its MDA content with increasing Al concentration [57]. However, the content of MDA in the roots of *Neolamarckia cadamba* (Roxb.) Bosser significantly decreases after three days of 400 µM Al treatment [59]. In our study, the MDA content initially increased and then decreased. With increasing Al concentrations, extra peroxidative injury was generated in peanut leaves due to Al poisoning, resulting in gradually increased malondialdehyde levels. However, when the concentration of Al reached its highest level, the MDA content significantly decreased. This could be attributed to severe damage to the defense system of antioxidants in peanut leaf cells at relatively high Al concentrations. Although the activities of CAT, SOD, APX, and POD increased with increasing Al concentrations, higher Al concentrations might disturb the internal processes of cells, thereby explaining the decrease in MDA content.

Compared with the control, the Al content in leaves of peanut markedly rose with the adoption of the Al poisoning treatment. This result was believed to be on account of peanut leaves being poisoned by aluminum. Previous research has indicated that Al tends to bind to unmethylated pectin in the cell wall, bringing about a large accumulation of Al in the cytoderm and increased rigidity [60]. This eventually affects cell division [60]. However, in rye treated with Al, upregulated expression of genes related to glycosylhydrolase has been observed [15]. These glucanases have been shown to be involved in the cell division process, and their upregulation may contribute to cell division under Al stress [15].

Additionally, the levels of K and Fe in Al-treated peanut leaves also significantly increased. Notably, previous studies have shown a significant decrease in the K content in peanut roots subjected to Al treatment [12]. This suggests that peanut plants transfer more K to leaves after suffering Al toxicity [61]. The supply of K can enhance the photosynthetic rate of leaves [61]. In rice, the application of K promotes the conversion of stored nitrogen in leaves into photosynthetic nitrogen, thereby improving the efficiency of photosynthetic nitrogen use in leaves [61]. Fe has an essential role in the biosynthesis of porphyrin rings of chlorophyll molecules and facilitates the structural integrity of the LHC (light-harvesting complex) subunits and photosynthetic reactivity centers [62]. Within plant cells, chloroplasts have the highest demand for Fe [63]. In *Hordeum vulgare* L. cv. Luch leaves, chloroplasts account for more than 90% of the total Fe content, highlighting their importance [63]. Under conditions of hydroponic iron deficiency, the chlorophyll content in wheat (*Triticum aestivum* L.) leaves decreased by 30% compared with the control, indicating the detrimental impact of iron deficiency on plant photosynthesis [62]. The experimental results of the present research revealed that peanuts may alleviate the negative effects of Al toxicity stress by accumulating relatively high levels of K and Fe in their leaves.

In plants, Al has antagonistic associations with Mg and Mn [64,65]. Magnesium is an essential macronutrient that has a key role in plant photosynthesis, energy metabolism, and nucleic acid and protein synthesis [66]. A deficiency in Mg severely impairs the light collection and carboxylation processes in rice, which are essential for photosynthesis [66]. On the other hand, Mn deficiency reduces the supply of electrons from PSII to PSI, leading to rapid suppression of the PSI parameter Y(NA) [67]. Ca, on the other hand, affects the fluidity of cell membranes and is an important component of pectin in plant cell walls [68]. Calcium ions, as a molecular signal of physiological regulation, can compete with Al^3+^ for binding sites in pectin [69]. In this study, the decrease in Mg, Mn, and Ca contents in peanut leaf cells could be due to the high Al^3+^concentration taking up the binding sites in the leaf cells. In sunflower (*Helianthus annuus* L.), Cu-deficient plants have a low content of photo-active plastocyanin [67]. Similarly, the decrease in Cu content in peanut leaves, mediated by Al, could also be one of the factors influencing leaf photosynthesis.

The genes of the natural-resistance-associated macrophage protein (NRAMP) family have crucial roles in the assimilation and transport of heavy-metal elements such as Cu, Zn, Mn, and Fe [70]. It is well known that they participate in plants’ response to heavy metal stress [70]. In potato (*Solanum tuberosum* L.), five NRAMP family genes have been identified, among which *StNramp2* has the highest expression level in leaves. After exposure to excessive amounts of Cu, Zn, Ni, Pb, and Cd, the expression level of *StNramp2* in leaves significantly increases [70]. This suggests that *StNramp2* has great potential for responding to metal stress in potato plants [70]. The MTP (plant metal tolerance protein) family of genes is important for enhancing plant resistance to heavy metals and maintaining the metal ion balance in plants [71]. In tomato, eleven MTP family members have been identified, and the expression of *SlMTP4* in tomato leaves significantly increases upon treatment with Cd, Co, Fe, Mn, and Zn [71]. This indicates that *SlMTP4* has a crucial part in the response of tomato to metal stress [71]. In this research, some members of the AhMTP and AhNRAMP families displayed differential gene expression in peanut leaves subjected to Al toxicity. Those results suggest that the AhMTP and AhNRAMP families might also play key roles in peanut tolerance to Al toxicity. Previous studies have focused primarily on the transportation of divalent ions such as Fe^2+^ and Cd^2+^ by the NRAMP and MTP families [33,35,70]. Based on the experimental results of this study, it could be hypothesized that the AhMTP and AhNRAMP families might also be involved in the transportation of Al^3+^.

ABA is one of the crucial phytohormones in plant resistance to metal poisoning [72]. Results have demonstrated that the transcription factor *NGAATHA1* in *Arabidopsis thaliana* positively adjusts the production of ABA under abiotic stress by stimulating NCED3, which encodes a pivotal ABA synthesis factor [73]. ABA is thought to be associated with the stomatal conductivity of leaves [30]. When subjected to Al poisoning, the ABA level of lemon (*Citrus × limonia* Osbeck (pro. Sp.)) significantly increases after just one day, indicating that Al toxicity induces ABA synthesis in leaves [30]. As a result, leaf water potential, stomatal conductance, and overall plant transpiration are affected [30]. This study suggested that increasing peanut leaf ABA levels by upregulating the expression level of related genes might reduce the negative consequences of high concentrations of Al on plant aquation.

Zeatin, a natural plant cytokinin, influences leaf size by controlling the division and expansion of leaf cells [74]. When transgenic *Arabidopsis thaliana* (L.) Heynh. plants harboring the dexamethasone (DEX)-induced *ipt* gene are treated with DEX, the cytokinin content increases excessively, resulting in a significant decrease in the average number of leaf cells [75]. It was noted that following Al poisoning, the Zeatin content decreased significantly in peanut leaves. This decrease might be due to Al induction affecting the expression level of related genes in leaves of peanut and subsequently inhibiting the cell division cycle and development in peanut leaves.

The IAA-mediated TIR1 (transport inhibitor response 1)-ARF (Aux/IAA-auxin response factor) pathway is a well-known auxin signal transduction pathway [76]. Among the key factors in this pathway is ARF [76]. By infecting *Arabidopsis thaliana* (L.) Heynh. with *Agrobacterium tumefaciens*, the over-expression of the *CgARF1* from *Cymbidium goeringii* ‘Songmei’ induces leaf aging, increases the root number in vitro, and causes variations in phytohormone contents after dealing with IAA [76]. These findings indicate that CgARF1 is involved in regulating leaf growth through IAA [76]. In the present study, the increased IAA content may have affected the growth of peanut leaves.

SA plays a role in regulating the adversity stress response in plant and accelerates the production of ROS via antioxidative defense systems [77,78]. When subjected to salt stress, exogenous SA application helps to enhance leaf photosynthesis in cucumbers (*Cucumis sativus* L.), thereby alleviating salt stress [79]. SA-binding protein 2 (SABP2) possesses salicylate methyl ester activity. A gene similar to *SABP2*, *LcSABP*, was identified in *Lycium chinense* Miller [80]. Compared with the control, the presence of *LcSABP* in tobacco markedly elevated the endogenous SA content [80]. Studies have shown that when *Fagopyrum esculentum* Moench. is subjected to aluminum toxicity stress, endogenous ABA levels and antioxidant enzyme activities increase synchronously [81]. This is because plants enhance antioxidative enzyme activities by increasing the endogenous ABA levels [81]. Therefore, actively increasing ABA content may also be one of the means for plants to cope with abiotic stress [81]. Other studies have shown that exogenous SA treatment can reverse the accumulation of pectin in the root cell wall of Sanqi (*Panax notoginsen* (Burk.) F.H.) caused by aluminum stress and decrease the degree of pectin methylation, thus reducing the content of aluminum in pectin in Sanqi [82]. Therefore, the increase in SA and ABA content after aluminum toxicity stress in this study might be an effective way to resist aluminum in peanut plants. Thus, this study suggests that an elevated SA content in peanut leaves may facilitate photosynthesis and serve as one of the mechanisms by which peanuts alleviate Al toxicity stress.

JA can help plants adapt to stress [83]. After exposure to aluminum, the application of exogenous methyl jasmonate (MeJA) can mitigate the damage caused by aluminum toxicity stress in blueberry (*Vaccinium corymbosum* L.) [83]. The heterologous expression of the *rolB* and *rolC* genes from sweet potato (*Ipomoea batatas* (L.) Lam.) significantly enhances the production of MeJA in transgenic *Arabidopsis thaliana* (L.) Heynh., suggesting the involvement of Ib-rolB and Ib-rolC in the biosynthesis of JA [84]. In this study, the notable rise in JA levels observed in the tablets might be a mechanism to reduce the harm caused by excessive aluminum ions.

GA_3_ has an important effect in facilitating seed germination and leaf development [85]. The curly leaves of lettuce (*Lactuca sativa* L.) are affected by the *LsKN1* hormone-regulating gene, and upregulation of *LsKN1* leads to a decrease in gibberellin biosynthesis, resulting in reduced gibberellin content and flattened leaves in lettuce [29]. In this study, it is likely that the expression level of genes participating in the synthesis of GA_3_ in peanut leaves is affected by Al toxicity stress, resulting in a significant decrease in the GA_3_ content.

The DEGs observed in this study can mainly be classified into three types: physiological response genes (*SOD2*, *CAT1*, *APX1*, and *POD1*), genes associated with ion transport (*Nramp3.1*, *Nramp5*, *Nramp6.2*, *MTP1.2*, *MTP4.1*, *MTP10*, and *MTPB1*), and genes associated with hormones (*ABA1*, *IAA1*, *SA2*, *Zeatin1*, and *GA3.1*). The role of genes related to physiological responses and hormones is crucial for regulating the plant response to environmental stress and for identifying ROS [86]. The differentially expressed genes involved in physiological responses and hormones under Al stress indicate that plants can withstand stress by stimulating those genes and eliminating superabundant ROS in leaves, thereby coping with aluminum toxicity stress [86]. The NRAMP family has an important role in the assimilation and transport of metals in plant [70], whereas MTPs are indispensable for the transport of divalent cations in plants [71]. Previous studies have connected the MTP and NRAMP family to the transport of divalent ions such as Fe^2+^ and Cd^2+^ [70,71]. In this research, some *AhMTPs* and *AhNRAMPs* exhibited differential expression in aluminum-poisoned peanut leaves, revealing that the AhMTP and AhNRAMP families might also have an important role in peanut tolerance to aluminum poisoning stress. In addition, those transporters relevant to divalent metallic ions might participate in the transportation of trivalent Al^3+^, offering new insights into how peanuts tolerate excess aluminum.

**Figure 8:**
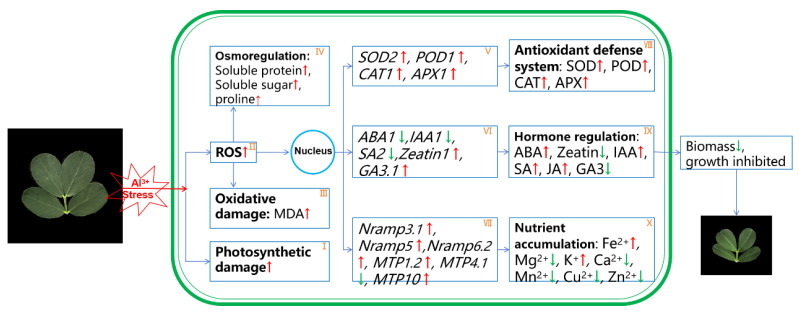
Regulation pathway showing how peanut leaves respond to aluminum toxicity. (**I**) Photosynthetic damage; (**II**) Reactive oxygen species; (**III**) Oxidative damage; (**IV**) Osmotic conditioning; (**V**) Genes related to antioxidative enzyme activity; (**VI**) Plant hormone-related genes; (**VII**) Ion transport-related genes; (**VIII**) Antioxidative defense system; (**IX**) Hormone content; (**X**) Accumulation of mineral nutrient elements. Small red arrows indicate increased substance content or increased enzyme activity. Small green arrows indicate reduced enzyme activity and reduced or inhibited substance content.

Based on the experimental results of this study, Figure 8 illustrates the physiological regulation mechanism of peanut leaves in response to Al poisoning. Initially, when exposed to high levels of external Al, an increased amount of Al is transported to the leaf blade. This excess Al decreases the photosynthetic capacity of plants, leading to a rise in ROS and oxidative damage in leaf cells (Figure 8I–III). To maintain stability, cells respond by regulating their internal environment via osmotic adjustment substances (Figure 8IV). High concentrations of Al activated the mechanisms of cellular defense, bringing about the differential expression of genes coding antioxidative enzymes (Figure 8V). In the end, the activity of antioxidative enzymes was increased, efficiently removing superfluous reactive oxygen species (Figure 8VIII). In the presence of high Al concentrations, the differential expression of genes involved in phytohormone- (Figure 8VI) and ion transport-related genes (Figure 8VII) led to changes in plant hormones (Figure 8IX), ultimately reducing the ability of the leaves to accumulate mineral nutrients (Figure 8X). Ultimately, these changes resulted in a decrease in the biomass of peanut leaves.

## 4. Materials and Methods

### 4.1. Plant Materials and Disposal

The peanut variety used in this study was Zhanyou 62, a cultivar selected and bred by the Zhanjiang Institute of Agricultural Sciences in Zhanjiang, Guangdong Province, China. The seeds were then planted in the experimental field of Guangdong Ocean University for further propagation. The experimental materials were cultured in the greenhouse at the Coastal Agricultural Sciences College in GDOU (Guangdong Ocean University; eastern longitude: 110.30311, northern latitude: 21.15005). Peanut seeds of similar size and full maturity were carefully selected and disinfected using 1% sodium hypochlorite (Guangzhou reagent, Guangzhou, China) for 10 min. These sterilized seeds were subsequently germinated in sterilized white quartz sand for a period of 10 days. After germination, uniformly growing peanut plants were transferred to 15 L blue plastic boxes for hydroponic cultivation. A nutrient solution according to [87] was added to the boxes. The composition of the nutrient solution is detailed in Table S1. All of the chemicals applied in the experiment were analytically pure and were obtained from Solarbio, Beijing, China. Al_2_(SO_4_)_3_·18H_2_O (Guangzhou reagent, Guangzhou, China) was adopted as exogenous Al, and varying different concentrations of Al^3+^ (0, 0.5, 1, 2, 4, and 8 mM) were added to the nutrient solution for treatment. The control was dealt with 0 mM Al^3+^. Every experiment was repeated three times. The number of peanut plants in each treatment group was 24–30. The temperature was regulated between 25–27 °C during the day and 19–22 °C at night to ensure optimal plant development. The photoperiod was set at approximately 12 h/d. The nutrient solution was replaced 4 days apart. Peanut leaves (from the fourth leaf position) from each Al treatment group were collected after 20 days of culture.

### 4.2. Measurement of the Dry and Fresh Weights of Peanut Leaves

The fourth leaf from each peanut plant was harvested post-treatment, and its fresh weight was accurately weighed via an electronic analytical balance (Sartorius BS124S, Gottingen, Germany). Subsequently, the plant samples were placed in a constant-temperature drying box (Shanghai Yiheng, Shanghai, China) and baked at 60 °C for 7 days [88]. The dry weight of the peanut leaves was then tested separately.

### 4.3. Determination of the Peanut Leaf Morphology Index

Leaf physiognomy and lesion analysis (Topu Yunnong, Hangzhou, Zhejiang, China) were employed to record the morphological characteristics of peanut leaves, and pictures of each Al-treated peanut leaf were taken. The data regarding the total area and total perimeter of the leaves were analyzed. These measurements were repeated three times.

### 4.4. Determination of Chlorophyll Content

The content of chlorophyll and total carotenoids was determined and calculated following Chen’s method [89]. After 20 days of treatment at different Al^3+^ concentrations (0, 0.5, 1, 2, 4, and 8 mM), approximately 0.2 g of fresh leaf sample from the fourth leaf of chopped peanut plants was taken and placed into a glass test tube containing 10 mL of 95% ethanol (Solarbio, Beijing, China). Chlorophyll-a, chlorophyll-b, and total carotenoids were extracted by soaking for 24 h in completely dark conditions until the leaves became completely white. The extracts were then detected using an ultraviolet spectrophotometer (Shanghai Yuexi UV-5100B, Shanghai, China) at 470 nm, 649 nm, and 665 nm. Each treatment was replicated three times.

### 4.5. Determination of the Physiological Response Indices of Peanut Leaves

The peanuts were sequentially treated with Al^3+^ at concentrations of 0, 0.5, 1, 2, 4, and 8 mM. After 20 days, leaf material from each treatment were collected, and eight kinds of physiological indices were tested. The MDA content was measured using the thiobarbituric acid method [90]. The activity of POD was tested using the guaiacol method [91]. The activity of CAT was measured using a spectrophotometer [92]. APX activity was measured using Li’s experimental method [93]. The proline (Pro) content was tested via the sulfosalicylic acid method [94]. The content of soluble protein was tested by Coomassie brilliant blue staining [95]. The NBT (nitro blue tetrazolium) method was employed to determine the activity of SOD [96]. The content of soluble sugars was determined using the anthrone method [97].

### 4.6. Determination of Al, Na, K, Mg, Mn, Fe, Cu, Zn, Ca, and Se Contents in Peanut Leaves

Peanuts were treated with 0 or 4 mM Al^3+^, and the treated leaf material was collected after 20 days. The treated samples were placed in a constant-temperature drying box (Shanghai Yiheng, China) and baked at a constant temperature of 60 °C for 7 days. Subsequently, the samples were weighed to 0.15 g for each treatment and completely dissolved in nitric acid. Subsequently, the samples were weighed to 0.15 g per treatment in a tetrafluoroethylene crucible (30 mL) (Puqi, Yancheng, Jiangsu, China), and then 5 mL of 98% concentrated nitric acid (Huada Corporation, Guangzhou, Guangdong, China) was added and left for 24 h. The crucible was moved to the heating plate (Richen LC-DB-3EFS, Shanghai, China), and the temperature was gradually raised to 180 °C for digestion for 2~3 h. After cooling, 2 mL nitric acid (98%) and 2 mL hydrogen peroxide (30%) (Huada Corporation, Guangzhou, Guangdong, China) were added to the tetrafluoroethylene crucible, which was placed on the electric heating plate at 180 °C to digest for 3~5 h. After cooling the sample, it was heated at 140 °C until it was nearly dry. After cooling to room temperature, 1 mL of nitric acid (98%) was added to leach the sample, and then all the extract and residue were transferred into a 25 mL colorimetric tube (Zhihong, Taizhou, Jiangsu, China) containing deionized water. The volume was adjusted to the scale line with deionized water, and the supernatant was taken for subsequent ion content measurement after standing. The leaf samples were then measured via ICP–AES (inductively coupled plasma–atomic emission spectrometry) (Hitachi PS7800, Tokyo, Japan) to determine the levels of Na, Mg, Mn, K, Fe, Se, Cu, Ca, Zn, and Al in peanut leaves [86,98]. Each index was replicated three times.

### 4.7. Determination of the Levels of Six Phytohormones in Peanut Leaves 

The peanuts were treated with Al^3+^ at concentrations of 0 or 4 mmol/L, and the treated leaf material was collected after 20 days. HPLC (high-performance liquid chromatography) (AGLIENT1290, Palo Alto, California, USA) combined with tandem mass spectrometry (MS/MS; AB SCIEX-6500Qtrap, Milwaukee, Wisconsin, USA) was used to extract the plant endogenous hormones and determine their content in peanut leaves. The hormones analyzed included IAA, ABA, SA, Zeatin, JA, and GA_3_. An internal reference substance was also added to the extract to ensure accurate determination of the hormone content [99]. The external standards used in the analysis, such as IAA, ABA, SA, Zeatin, JA, and GA_3_, were pure chromatographic reagents purchased from Sigma (Saint Louis, MI, USA). The interior labels used were deuterated versions of IAA (D-IAA), ABA (D-ABA), SA (D-SA), Zeatin (D-Zeatin), JA (D-JA), and GA_3_ (D-GA_3_) from Sigma. C18 QuECherS (Shanghai Amperex, Shanghai, China) was adopted as the filling material for the column, and chromatographic grade acetonitrile and methanol from Merck (Darmstadt, Germany) were used as solvents.

Liu et al. [95] investigated the preparation of the working solution, the calculation of the standard curve for hormone content, and the extraction of hormones from peanut leaves. Additional information, including the gradient parameters of the HPLC, the parameters of mass spectrometry, and selected monitoring conditions for protonated or deprotonated plant hormone reactions, can be found in Supplementary Table S2, Table S3, and Table S4, respectively.

### 4.8. qPCR (Quantitative RT‒PCR) Fluorescence Analysis

Several differentially expressed genes were confirmed in previous studies through peanut transcriptome sequencing, suggesting their potential involvement in antioxidative properties and hormone regulation in peanuts under metal ion stress [88]. Therefore, we hypothesized that these genes hold research value for studying peanut resistance to aluminum toxicity. To investigate the possible function of those genes in the response of peanut leaves to Al toxicity stress, their expression was verified using qPCR. Peanuts were subjected to treatment with 0 or 4 mmol/L Al^3+,^ and the leaves were collected after 20 days of hydroponic cultivation. RNA was extracted from the peanut leaves using the MolPure Plant RNA Kit (Yeasen, Shanghai, China), followed by reverse transcription to obtain cDNA using the Hifair II 1st Strand cDNA Synthesis Kit (Yeasen, China). The expression of target genes was detected using the Hieff UNICON Universal Blue qPCR SYBR Green Master Mix (Yeasen, Shanghai, China) and ABI7500 fluorescence quantitative PCR (Applied Biosystems, Carlsbad, CA, USA) [100].

The expression levels of genes participating in hormone synthesis, ion transport, and physiological responses in peanut leaves were analyzed by qPCR. The qPCR system included 20 μL of reaction mixture, which consisted of 8 μL of nuclease-free water, 1 μL of cDNA template, 0.5 μL of the downstream primer, 0.5 μL of the upstream primer, and 10 μL of the Hieff UNICON Universal Blue qPCR SYBR Green Master Mix (Yeasen, China). The qPCR procedure consisted of an initial denaturation step at 95 °C for 10 min, followed by 40 cycles of denaturation at 95 °C for 15 s and annealing/extension at 60 °C for 1 min [101]. The peanut cDNA template obtained from reverse transcription was appropriately diluted and used as standard product 1. Standard 1 was further diluted five times to obtain standard 2. This dilution process was repeated to generate standard 3, standard 4, and standard 5 for generating the standard curves. The *AhUbiquitin* (*DQ887087.1*) was selected as the reference gene, and the relative transcript levels of the target genes were calculated using the following equation: relative gene transcript level = target gene transcript level/internal reference gene expression level [102]. The primers used in the qPCR in this study are displayed in Table S5.

### 4.9. Data Analysis

Microsoft Excel 2010 (Microsoft, Redmond, WA, USA) was adopted for statistical analysis, whereas IBM SPSS Statistics software (version 26) was used for data analysis. Independent sample *t* tests were performed to compare and determine the significance between two groups of data. For multiple sets of data, Waller–Duncan’s test from the single-factor ANOVA test in the software was used for the multiple comparison of data and significant difference analysis.

## 5. Conclusions

On the basis of the experimental findings of this research, the physiological response mechanism of peanut leaves to aluminum poisoning was revealed. Firstly, the presence of a substantial amount of exogenous Al during Al poisoning stress resulted in an evident rise in the transport of Al to the peanut leaves. Consequently, this increase in Al content triggered the activation of cellular defense mechanisms, resulting in changes in the expression levels of genes encoding antioxidative enzymes and the enhancement of antioxidative enzyme activity. This dynamic response effectively eliminated excessive ROS. Furthermore, the existence of excessive Al ions in the leaves induced the expression of genes relevant to phytohormones and ion transport. These changes led to variations in the expression of genes participating in the transport of plant hormones and metal ions, subsequently impeding the accumulation of various nutrients in the leaves. Ultimately, this cascade of events results in symptoms that include reduced leaf biomass, restricted leaf growth, and aberrant aggregation of metallic ions. The findings of this research have laid the groundwork for further investigations into the physiological function and molecular regulation mechanisms underlying the response of peanut leaves to Al toxicity. Additionally, these results provide a theoretical foundation for breeding endeavors aimed at developing new Al-resistant varieties of peanuts. Furthermore, this study only takes plant physiological responses, ion accumulation, and hormone regulation as the starting point, and the research field is not broad enough. In future studies, a combined multi-omics method can be used to study the process of the peanut leaf response to aluminum toxicity stress in more detail. At the same time, key genes will be mined, focusing on the specific molecular mechanisms of functional genes involved in regulating the leaf response to aluminum toxicity stress.

## Data Availability

Data are contained within the article and Supplementary Materials.

## Supplementary Materials

The following supporting information can be downloaded at: https://www.mdpi.com/article/10.3390/plants13121606/s1, Table S1: Composition and content in hydroponic solution. Table S2: The detailed gradient parameters for HPLC; Table S3: Mass spectroscopy parameters; Table S4: Selected monitoring indicators for the protonation or deprotonation reactions of phytohormones; Table S5: The primers used for qPCR in this study.
